# The first complete mitochondrial genome of *Euroleon coreanus* (Okamoto, 1926) (Neuroptera: Myrmeleontidae) and its phylogeny

**DOI:** 10.1080/23802359.2021.1937362

**Published:** 2021-06-14

**Authors:** Jia-Yin Guan, Hua Zhang, Zi-Yi Zhang, Yu-Rou Cao, Kenneth B. Storey, Jia-Yong Zhang, Dan-Na Yu

**Affiliations:** a College of Chemistry and Life Science, Zhejiang Normal University, Jinhua, Zhejiang Province, China; b School of Chemical Engineering, Liaoyang Vocational College of Technology, Liaoyang, Liaoning Province, China; c Department of Biology, Carleton University, Ottawa, Canada; d Key Lab of Wildlife Biotechnology, Conservation and Utilization of Zhejiang Province, Zhejiang Normal University, Jinhua, Zhejiang Province, China

**Keywords:** *Euroleon coreanus*, Myrmeleontidae, mitogenome, phylogenetic relationship

## Abstract

The first complete mitochondrial genome of *Euroleon coreanus* (Okamoto, 1926) was 15,797 bp in length, and contained 13 protein-coding genes, 22 transfer RNAs, two ribosomal RNAs, and the control region. Compared to the classic insect mitochondrial genome, *E. coreanus* showed a gene rearrangement of *ND2-C-W-Y-COX1*. The overall AT content of the mitochondrial genome was 75.5%. The monophyly of Ascalaphidae, Myrmeleontidae, Nemopteridae, Nymphidae, and Psychopsidae was supported in both BI and ML trees. And *E. coreanus* was a sister clade to the clade of genus *Myrmeleon*.

Neuroptera is an early-diverging lineage of holometabolous insects including about 6,000 species in 19 families (Song et al. 2019; Oswald 2020). Myrmeleontidae is one of the most species-rich families within Neuroptera (Machado et al. 2019; Machado and Oswald 2020; Zheng and Liu 2021) The phylogenetic relationship between Myrmeleontidae and Ascalaphidae was controversial in past studies that were based on morphological and molecular data (Aspöck et al. 2001; Winterton et al. 2010; Yan et al. 2014; Wang et al. 2017; Zhang and Yang 2017; Song et al. 2018; Winterton et al. 2018). *Euroleon coreanus* (Okamoto, 1926) is currently assigned to the subfamily Myrmeleontinae of Myrmeleontidae, which is widely distributed in Korea, Mongolia, and North China (Liu et al. 2016). Besides, the larvae can be treated as traditional Chinese medicine (Liu et al. 2016). In this study, we sequenced the complete mitochondrial genome of *E. coreanus* to enrich the molecular data of Myrmeleontidae, and further discuss the phylogenetic status of Myrmeleontidae.

The sample of *E. coreanus* was collected from Yitong Manchu Autonomous County (N 43.200°, E 125.170°), Jilin Province, China, then identified by Dr. JY Zhang. The specimen under the voucher number: JLYT20190826-1 was preserved in a −40 °C freezer and deposited at the Animal Specimen Museum, College of Life Sciences and Chemistry, Zhejiang Normal University, China (sky.zjnu.edu.cn, DN Yu, ydn@zjnu.cn). Total DNA was extracted from muscle tissue of forelegs using an Ezup Column Animal Genomic DNA Purification Kit (Sangon Biotech Company, Shanghai, China) and stored in the Zhang laboratory. We used usual primers to amplify several partial fragments (Simon et al. 2006) and designed species-specific primers based on previously obtained sequences using Primer Premier 5.0 (Lalitha 2000). All PCR products were then sequenced using the primer-walking method by Sangon Biotech Company (Shanghai, China). The tRNA genes were identified using the MITOS web server (http://mitos.bioinf.uni-leipzig.de/index.py) (Bernt et al. 2013). Thirteen PCGs and two rRNA genes (*12S* and *16S rRNA*) were identified by aligning with homologous gene sequences of other Myrmeleontidae species using Mega 7.0 (Kumar et al. 2016). In addition, we translated the nucleotide sequences of the 13 protein-coding genes (PCGs) into amino acids based on the invertebrate genetic codes. The *E. coreanus* mitochondrial genome was deposited in GenBank with an accession number MW879174.

The complete mitochondrial genome of *E. coreanus* was 15,797 bp in length, including 13 protein-coding genes (PCGs), 22 transfer RNAs, two ribosomal RNAs, and a control region (982 bp). The nucleotide composition of *E. coreanus* was as follows: A = 39.2%, T = 36.3%, C = 14.6%, G = 9.9%. The overall AT content of the mitochondrial genome was 75.5%. Compared to the classic insect mitochondrial gene sequence, the *trnW* gene had moved into a position between *trnC* and *trnY*, which gave a gene order of *ND2-C-W-Y-COX1*. However, this gene arrangement is common in the mitochondrial genomes of Myrmeleontidae (Song et al. 2019). For the 13 PCGs, almost all used ATN as the initiation codon, excluding *COX1* that used ACG. *COX1*, *COX2* and *ND5* used stop codons that were incomplete (T-) whereas the remaining 10 protein-coding genes ended with typical stop codons (TAA or TAG). Four protein-coding genes (*ND1*, *ND4*, *ND4L*, *ND5*), eight tRNA genes (*tRNA^Gln^*, *tRNA^Cys^*, *tRNA^Tyr^*, *tRNA^Phe^*, *tRNA^His^*, *tRNA^Pro^*, *tRNA^Leu1^*, *tRNA^Val^*) and two rRNA genes (*16S rRNA*, *12S rRNA*) were located on the light strand (L strand), the remaining 23 genes were coded on the heavy strand (H strand). Overall, nine gene overlaps and 11 gene-spacing regions between adjacent genes were found in the mitochondrial genome of *E. coreanus*, ranging from 1 to 7 bp and 1 to 69 bp in length, respectively.

Based on the 13 PCGs, the phylogenetic relationship was constructed using two methods: Bayesian inference (BI) using MrBayes 3.1.2 (Huelsenbeck and Ronquist 2001) and Maximum-likelihood (ML) using RAxML 8.2.0 (Stamatakis 2014). The mitochondrial genomes of 21 Neuroptera species including *E. coreanus* and 20 species downloaded from GenBank were used to investigate the phylogenetic relationships within Neuroptera (Beckenbach and Stewart 2009; Negrisolo et al. 2011; Cheng et al. 2014; Yan et al. 2014; Cheng et al. 2015; Lan et al. 2016; Zhang and Wang 2016; Zhang and Yang 2017; Gao et al. 2018; Song et al. 2019; Wu et al. 2020), with *Xanthostigma gobicola* chosen as the outgroup. The results of the two phylogenetic analyses (BI and ML) using the 13 protein-coding genes yielded the same topological structure (Figure 1). The monophyly of Ascalaphidae, Myrmeleontidae, Nemopteridae, Nymphidae, and Psychopsidae was supported in both BI and ML trees. Nymphidae was the basal clade of Neuroptera and then the phylogenetic relationship of (Nymphidae + (Psychopsidae + (Nemopteridae + (Ascalaphidae + Myrmeleontidae)))) was strongly supported. In addition, *E. coreanus* was identified as a sister clade to the clade of (*Myrmeleon formicarius* + (*M. immanis* KJ461323 + *M. immanis* KM216750)).

**Figure 1.**
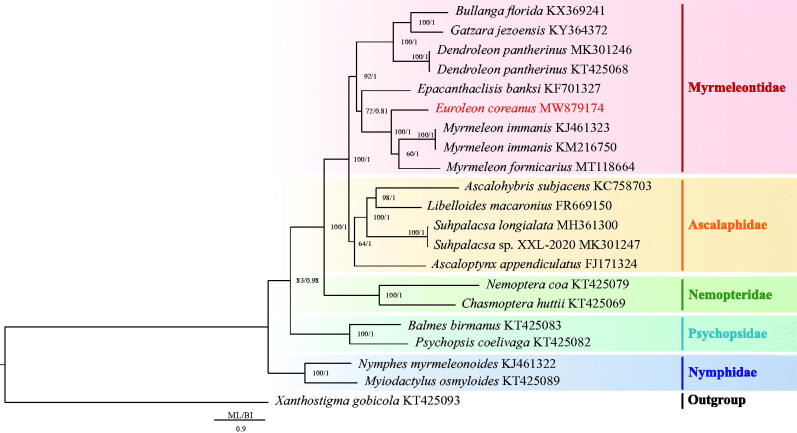
Phylogenetic tree of the relationships among 21 species of Neuroptera including *Euroleon coreanus.* The mitochondrial genomes of 20 Neuroptera species were downloaded from GenBank and used to investigate the phylogenetic relationships, with *Xanthostigma gobicola* chosen as the outgroup. Numbers around the nodes show the posterior probabilities of BI (right) and the bootstrap values of ML (left). GenBank numbers of all species are shown in the figure.

## Data Availability

The genome sequence data that support the findings of this study are openly available in GenBank of NCBI at (https://www.ncbi.nlm.nih.gov/) under the accession no. MW879174.
